# Risk factors associated with cardiac complication after total joint arthroplasty of the hip and knee: a systematic review

**DOI:** 10.1186/s13018-018-1058-9

**Published:** 2019-01-11

**Authors:** Yassin Elsiwy, Ivana Jovanovic, Kenji Doma, Kaushik Hazratwala, Hayley Letson

**Affiliations:** 1 Orthopaedic Research Institute of Queensland, Townsville, QLD Australia; 2 College of Medicine and Dentistry, James Cook University, Townsville, QLD Australia; 3 College of Healthcare Sciences, James Cook University, Townsville, QLD Australia

**Keywords:** Cardiac, Arthroplasty, TKA, THA, Risk factor, Complication

## Abstract

**Background:**

Cardiac complication represents a major cause of morbidity and mortality after total joint arthroplasty, thus necessitating investigation into the associated risks in total hip arthroplasty and total knee arthroplasty. There remains a lack of clarity for many risk factors in the current literature. The aim of this systematic review is to assess the most recent published literature and identify the risk factors associated with cardiac complication in total hip arthroplasty and total knee arthroplasty.

**Methods:**

Scopus, PubMed, CINHAL, and Cochrane were searched to identify studies published since 2008 reporting on risk factors associated with cardiac complication in elective primary in total hip arthroplasty and total knee arthroplasty in patients ≥18 years old with osteoarthritis. Reported odds ratios, hazard ratios, and relative risk were the principal summary measures collected. The included studies were too heterogeneous to enable meta-analysis.

**Results:**

Fifteen studies were included in this systematic review. Increasing age and history of cardiac disease were found by most studies to be positively associated with risk of cardiac complication. There was no strong association found between obesity and cardiac complication. The evidence for other risk factors was less clear in the examined literature, although there is suggestive evidence for male gender and cerebrovascular disease increasing risk.

**Conclusions:**

Increasing age and history of cardiac disease increases the risk of cardiac complication after total hip arthroplasty and total knee arthroplasty. Other risk factors commonly attributed to increased risk in non-cardiac surgery including hypertension and obesity require further evaluation in arthroplasty.

**Systematic review registration:**

A detailed protocol was published in the PROSPERO database (registration number CRD42018095887) for this systematic review.

**Electronic supplementary material:**

The online version of this article (10.1186/s13018-018-1058-9) contains supplementary material, which is available to authorized users.

## Introduction

Total hip arthroplasty (THA) and total knee arthroplasty (TKA) is the definitive surgical treatment for osteoarthritis (OA) of the hip and knee to improve quality of life, alleviate pain, and enhance function [1–3]. It is predicted that by 2030, there will be a 174% and 673% growth for THA and TKA respectively, with a projected 4 million total joint arthroplasties (TJA) annually in the USA [4–6]. Although TJA is considered a relatively safe procedure with reported rates of major cardiac complication as low as 0.2 to 0.8%, this risk becomes increasingly significant when considering the substantial increase in arthroplasties expected in the future [7–11]. Cardiac complication represents a major cause of morbidity and mortality after TJA and is associated with increased hospital mortality, increased length of stay, increased non-cardiac events, and increased health expenditure [7, 12–15]. Therefore, there is a growing imperative to understand the risks contributing to cardiac complications associated with THA and TKA.

With advances in medical care increasing life expectancy combined with sedentary lifestyles, the TJA population is increasing in age and comorbidity, particularly obesity and diabetes [16–18]. This poses an evolving challenge in TJA; to navigate the increasing rate of arthroplasty procedures in a population with greater cardiovascular risk. In anticipation of this, there has been a surge of literature in the last decade to identify patient risk factors that predispose to cardiovascular complication. Nonetheless, there remains a lack of consensus and clarity for many risk factors.

Previous systematic reviews have focussed on identifying peri- and post-operative complications associated with TJA [19–25]. Risk factors for one specific complication, venous thromboembolism, were discussed in a review by Zhang et al. [19]; however, to the best of our knowledge, there are no systematic reviews specifically exploring the risk factors which contribute to major cardiac complications in THA and TKA. Therefore, the aim of this systematic review is to assess the most recent published literature and identify the risk factors associated with cardiac complication in THA and TKA.

## Materials and methods

### Search strategy

This systematic literature review was performed in accordance with the preferred reporting items for systematic review and meta-analysis (PRISMA) guidelines (see Additional file 1) [26]. Prior to commencing the study, a detailed protocol was published in the PROSPERO database (registration number CRD42018095887). Four databases including Scopus, MEDLINE/PubMed, CINAHL, and the Cochrane Library were searched on 13th of May, 2018 for the following search terms: (heart OR myocard* OR cardi*) AND (arthroplasty OR “joint replacement” OR “knee replacement” OR “hip replacement”) AND (risk OR complication OR adverse OR problem OR event OR issue OR mortality OR morbidity) AND (factor* OR characteristic* OR predispos* OR predic* OR profile). The following MeSH terms were also included in our search: Arthroplasty, Cardiology, Orthopaedics, and Risk Factors. Manual searches were also performed through hand searching the reference lists of included articles.

### Study selection

This review included studies that explored specific risk factors for cardiac complication associated with elective primary THA and TKA in patients ≥18 years old. Studies involving other types of arthroplasty or concerning revision THA or TKA were excluded. Only studies published from 2008 onwards were considered, and studies were excluded if more than 50% of their study population were operated on prior to 2008. This review only included studies in which the indication for TJA was osteoarthritis and excluded those with fractures, inflammatory, or autoimmune disease (rheumatoid arthritis, ankylosing spondylitis, psoriatic arthritis etc.). Studies examining the risk of cardiac complication associated with medication exposures, such as the use of anti-thrombotic agents and beta-blockers prior to surgery, were also excluded for the purpose of this analysis.

Cardiac complication was defined in this review as cardiac arrest (CA), myocardial infarction (MI), or new-onset arrhythmia, occurring within 90 days of THA or TKA. Articles which reported on general outcomes such as increased length of hospital stay or major complication without exploring the specific associations between a risk factor and cardiac complication were excluded. Literature that was not available in the English language, studies involving animal models, and levels IV and V evidence papers were also excluded.

Titles and abstracts were screened by two independent investigators (YE, IJ), and following exclusions, full-text articles were reviewed for eligibility. If two investigators could not reach a consensus on inclusion, a third investigator (HL) was involved in the final determination.

### Data extraction

Data extraction was performed using a pre-defined excel spreadsheet, which was prepared and agreed upon by three independent authors (YE, IJ, HL). Extracted data included information on study characteristics, patient demographics (age, gender, procedure) and comorbidities (cardiovascular and non-cardiovascular), as well as incidence of cardiac complication in each study. Reported odds ratios (OR), hazard ratios (HR), and relative risk (RR) were the principal summary measures collected to determine the association between studied risk factors and cardiac complication. Only significant values will be reported in this review, with statistical significance set as *p* < 0.05. Data on the definitions of cardiac complication and the length of follow-up was also extracted. Where possible, sub-group analysis between TKA and THA was collected to compare these cohorts in the analysis. Following data extraction, the included studies were too heterogeneous and therefore a meta-analysis was not conducted.

### Study quality assessment

A quality assessment tool was specifically developed for this review based on two existing appraisal tools: the Newcastle–Ottawa Scale and the Evidence-Based Library Appraisal Checklist (Additional file 2: Table S1) [27, 28]. Our tool included 25 questions, which assessed study methodology, population selection, diagnosis, and reporting of cardiac complications, as well as the level of analysis between THA and TKA cohorts. Two authors (YE and IJ) who were blinded from study identifiers (title, author, and journal) conducted the quality assessment. Any discrepancies of two or more points led to review by a third author (HL) and discussion until consensus was achieved. All 25 questions were equally weighted and individual scores were calculated based on the number of ‘YES’ answers. The final score was calculated as a percentage of the number of answerable questions. Studies that scored < 80%, 80–89%, and > 90% were deemed of low, moderate, and high quality, respectively.

## Results

### Selection of included studies

**Fig. 1:**
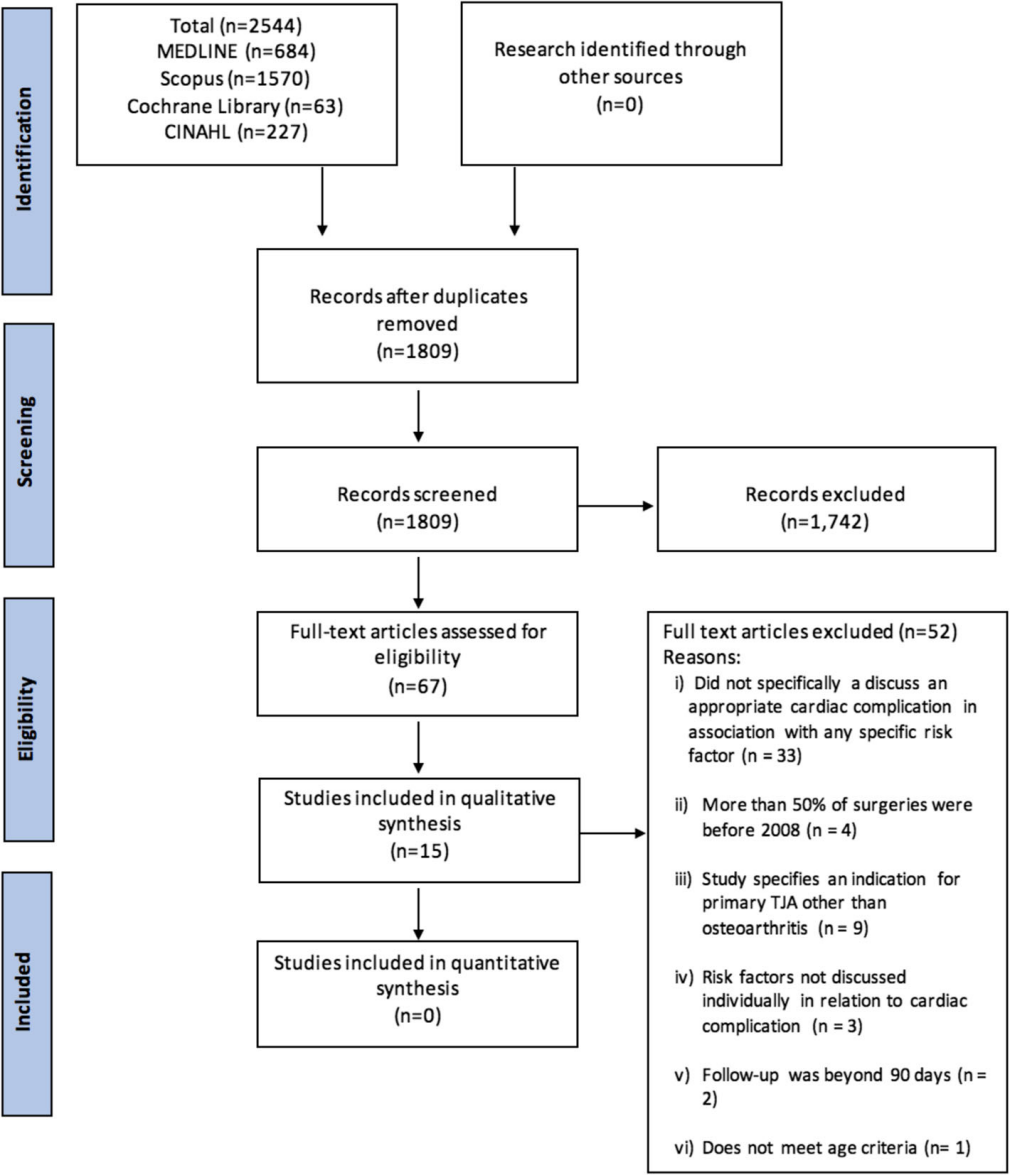
PRISMA flowchart. A total of 2544 studies were evaluated for an association between patient risk factors and cardiac complication. Titles and abstracts were assessed, and 67 full-text articles were eligible for evaluation. Fifty-two articles were excluded, and 15 articles remained for the final systematic review

The systematic search identified 2544 articles (Fig. 1). Following removal of duplicates, 1809 articles remained for screening. The majority of articles were excluded for arthroplasty other than primary THA or TKA (revision, shoulder, ankle, or elbow arthroplasty), indications other than osteoarthritis, and studies primarily concerned with infection or thrombo-embolic disease. Sixty-seven full-text articles were evaluated; 33 were excluded because they did not report any association between risk factors and cardiac complication, 9 included arthroplasties other than primary TJA for osteoarthritis, 4 in which > 50% of surgeries occurred prior to 2008, 3 for lack of specific risk factor analysis, and 3 for follow-up times beyond 90 days and population age outside of the inclusion range (Fig. 1). A total of 15 articles were eligible for inclusion in this systematic review, with a publication range from 2014 to 2018.

### Description of included studies

All studies included in this series were retrospective in nature (Table 1) with the exception of Abdel et al., which utilised a prospective design [29]. Fourteen studies were classified as level III evidence, reflecting the majority of cohort studies, whilst one was level II [29]. Many of the studies derived their study population from shared databases, such as the American College of Surgeons National Surgical Quality Improvement Program (ACS NSQIP) database, National Inpatient Sample (NIS), Medicare in-patient claims data, and other joint registries. A total of 8 of 15 articles included both THA and TKA study populations, 6 used TKA cohorts and 1 used THA (Table 1). Five studies included only unilateral TJA, three included both unilateral and bilateral procedures, and seven studies did not specify between unilateral and bilateral procedures.

**Table 1:** Study characteristics

	Year	Country	Range of data set	Evidence level	Data source	Unilateral vs. bilateral	Cardiac complication studied	Follow-up period	Main risk factors studied
Single cohort (TKA or THA) studies									
Fu [33]	2017	USA	2005–2 013	III	NSQIP	N/R	CA or MI	30 days	BMI and albumin concentration
Curtis [32]	2018	USA	2008–2014	III	NSQIP	N/R	CA or MI	30 days	Heart failure
Chamieh [35]	2016	USA	2008–2012	III	NSQIP	Unilateral	CA or MI	30 days	Pre-operative anaemia
Godoy [31]	2016	USA	2013–2015	III	Oschsner Clinic	Unilateral	MI or arrhythmia	90 days	Pre-operative ESR and CRP levels
Abdel [29]	2014	USA	2007–2010	II	Joint Replacement Registry	Unilateral	MI	I/H	BMI
Meller [34]	2016	USA	2011–2013	III	Medicare inpatient claims	Unilateral and bilateral	MI	90 days	BMI
Meller^γ^ [17]	2016	USA	2010–2014	III	Medicare inpatient claims	Unilateral and bilateral	MI	90 days	BMI
Combined (THA and TKA) cohorts									
Waterman [12]	2016	USA	2012–2013	III	NSQIP	Unilateral	MI and CA (requiring CPR)	30 days	Age, hypertension and cardiac disease
Robinson [6]	2017	USA	2012–2013	III	NSQIP	N/R	MI or CA	30 days	Gender
Feng [4]	2018	China	2005–2015	III	Hospital Joint Arthroplasty Registry	Unilateral and bilateral	MI or arrhythmia	30 and 90 days	Coronary artery disease and revascularisation
Thornqvist [8]	2014	Denmark	2005–2011	III	Danish National Patient Register and Danish Anaesthesia Register	N/R	MI or cardiac mortality	30 days	BMI
Menendez [11]	2015	USA	2008–2011	III	NIS	N/R	MI	I/H	Elixhauser comorbidity index variables and CAD, COPD, CVA, dementia
Shah [7]	2017	USA	2011–2014	III	NSQIP	N/R	MI or CA	30 days	TKA and THA
Belmont [5]	2014	USA	2006–2011	III	NSQIP	Unilateral	MI or CA	30 days	Many medical comorbidities and patient characteristics
Anoushiravani [30]	2016	USA	2006–2012	III	NIS	N/R	Cardiac complications	I/H	Underweight patients

Study characteristics were collected for each included study, including publication year, country of origin, and data source. Unilateral procedures were distinguished from bilateral procedures when possible and the period of follow-up post-operatively specified. The range of data set encompasses the period in which included arthroplasty procedures were performed, and the specific cardiac complication studied was also included. *CA* cardiac arrest, *MI* myocardial infarction, *CPR* cardiopulmonary resuscitation, *BMI* body mass index, *N/R* not reported, *NSQIP* National Surgical Quality Improvement Program database, *NIS* Nationwide Inpatient Sample, *CAD* coronary artery disease, *COPD* chronic obstructive pulmonary disease, *CVA* cerebrovascular disease, *I/H* in-hospital follow up (prior to discharge). γ = This is the only single cohort study that examined THA; the remainder of single cohort studies consisted of TKA

Seven studies followed patients for 30 days after TJA, four included a 90-day postoperative follow-up period, and four only reported in-hospital complications (Table 1). MI was assessed by 14 studies, CA in 7, and arrhythmia in only 2. Authors in one study [30] did not specify which cardiac complication they evaluated. Risk factors evaluated by the included studies included modifiable and non-modifiable patient factors, comorbidities, and surgery type (Table 1).

Across the 15 studies, study populations ranging in age between 18 and > 85 years old were included; most studies reported an average age of mid-to-late 60s (Table 2). The total number of study participants cannot be accurately identified due to a high likelihood of duplicate study populations. In 13 studies, TJA cohorts were predominated by the female gender (Table 2), whilst two studies did not provide gender-specific information of the total cohort in their primary text [5, 31]. The most common arthroplasty procedure in this series was TKA, with the exception of two studies [17, 30].

**Table 2:** Study demographics

Author	Total number of participants	Age range (years)	Female (%)
Single cohort (TKA or THA) studies			
Fu [33]	34,800	18 to > 80	63.2%
Curtis [32]	111,624	N/R	Control 63% Heart failure 55%
Chamieh [35]	41,334	18 to > 80	No anaemia 64.1% Any anaemia 62.5%
Godoy [31]	317	40 to 91	N/R
Abdel [29]	4718	Not specified	Obese 65.7% Non-obese 54%
Meller (TKA) [34]	585,127	65 to > 85	Normal weight 62% Morbidly obese 75% Super obese 82%
Meller (THA) [17]	432, 841	65 to > 85	Normal 62% Morbidly obese 68% Super obese 77%
Combined cohort (TKA and THA) studies			
Waterman [12]	34,066	N/R	TKA 63% THA 56%
Robinson [6]	54,502	18 to > 75	TKA 62.3% THA 54.9%
Feng [4]	4414	38 to 82	72%
Thornqvist [8]	34,744	59 to 82	59%
Menendez [11]	3,096,791	< 45 to > 85	No AMI 60.5% AMI 49.5%
Shah [7]	45,943	N/R	THA 49% TKA 53%
Belmont [5]	46,322	N/R	THA + THA 60.5%
Anoushiravani [30]	4865	N/R	THA Normal 85.7% Underweight 85.3% TKA Normal 78.0% Underweight 78.5%

The number of participants in each study was included; however, it was not possible to calculate the total number of participants across studies due to a high likelihood of duplicate studies (since many studies used similar databases). In the majority of studies, an age range of the study participants was specified; study populations ranging between 18 and > 85 years old were included in this review. Female gender formed the majority of participants across most study groups. *N/R* not reported; *AMI* acute myocardial infarction

### Risk of bias of the included studies

The inter-observer agreement on the quality of included studies ranged between 90 and 100%. Out of 15 articles included in this review, only 2 were deemed high quality, 11 were moderate quality, and 2 were low quality according to the appraisal tool used (Additional file 2: Table S1). Study quality was mainly limited by the use of study populations derived from large shared databases, the lack of specificity surrounding TJA details (indication, unilateral vs. bilateral), and failure to discuss definitions of the studied cardiac complication in relation to an accepted guideline.

### Association between risk factors and cardiac complication

The incidence of cardiac complication ranged from 0.07 to 3.0% across the studies [4, 7]. Several comorbidities were investigated for an association with cardiac complication in THA and TKA. Age, gender, type of arthroplasty, history of cardiac disease, hypertension, chronic pulmonary disease, diabetes, and obesity were thoroughly evaluated in our series (Table 3). Other risk factors including renal failure, malnutrition, anaemia, alcohol abuse, cerebrovascular disease, and smoking history were discussed, though reported to a lesser extent (Table 3).

**Table 3:** Number of studies examining each risk factor and the proportion of positive, negative, and insignificant associations found

Risk factors	Studies reporting on a risk factor for cardiac complication after THA or TKA			
	*N*	*p* ≤ 0.05		*p* > 0.05
		+	−	~
Age	6	4	−	2
Male	6	2	−	4
Female	6	–	1	4
TKA	4	1	−	2
THA	4	2	−	2
Unilateral	−	–	−	−
Bilateral	1	1	−	−
History of cardiac disease	5	4	−	1
CHF	4	2	−	2
Valvular disease	1	1	−	−
PVD	3	1	−	2
CAD	3	2	−	1
Hypertension	4	2	1	1
Chronic pulmonary disease	4	–	1	3
Diabetes	4	2	−	2
Renal failure	4	1	−	3
Obesity	8	1	−	7
Malnutrition	1	1	−	−
Anaemia	3	1	−	2
Alcohol abuse	1	−	1	−
CVD	4	2	−	2
Smoking history	1	1	−	−
ASA score > 2	2	1	−	1

The number of studies examining each risk factor and their association with cardiac complication in THA and TKA, and the number which showed a significant (*p* ≤ 0.05) positive and negative association. Studies, which studied a given risk factor but did not show any significant association (*p* > 0.05), are also identified. *CHF* congestive heart failure, *PVD* peripheral vascular disease, *CAD* coronary artery disease, *CVD* cerebrovascular disease, *ASA* American Society of Anaesthesiologists. *N* number of studies examining this risk factor; + studies showing a positive and significant association; − studies showing a negative, significant association; ~ insignificant result

### Age

Six studies reported on the relationship between age and cardiac complication (Additional file 3: Table S2) [4, 5, 7, 8, 11, 12]. For age ≥ 80 years, both Waterman et al. and Belmont Jr. et al. reported increased odds of cardiac complication in both TKA (OR 1.85; 95% CI 1.23–2.79; *p* = 0.003 and OR 27.95; CI 95% 2.01–388.93; *p* = 0.0016, respectively) and THA (OR 4.39; 95% CI 2.29–6.61; *p* < 0.001 and OR 3.72; CI 95% 1.53–9.06; *p* = 0.0001, respectively) [5, 12]. Menendez et al. similarly reported an increased risk for patients aged 45–64 (OR 4.4; CI 95% 2.6–7.4; *p* < 0.001), 65–84 (OR 6.5; CI 95% 3.8–11.0; *p* < 0.001), and > 85 (OR 9.4; CI 95% 5.5–16.1; *p* < 0.001) [11]. Conversely, Feng et al. did not show any association between age and cardiac complication (Additional file 3: Table S2) [4].

### Gender

The influence of gender on the risk of cardiac complication in TJA was also assessed in six studies (Additional file 3: Table S2) [4–8, 11]. Robinson et al. found that female gender was a protective factor for myocardial infarction in both THA (OR 0.54; 95% CI 0.40–0.75; *p* < 0.001) and TKA (OR 0.59; 95% CI 0.45–0.76; *p* < 0.001) [6]. In contrast, male gender was associated with increased risk of MI in THA (OR 1.84; 95% Cl 1.34–2.52; *p* < 0.001) and TKA (OR 1.71; 95% Cl 1.31–2.22; *p* < 0.001). Menendez et al. noted similar findings for male gender as a risk (OR 1.4; CI 95% 1.4–1.5; *p* < 0.001) [11]. Feng et al., Shah et al., and Belmont Jr. et al. did not find a statistically significant predictive value of gender for cardiac complication post-TJA (Additional file 3: Table S2) [4, 5, 7].

### History of cardiac disease

Seven articles explored the relationship between a history of cardiac disease and post-operative cardiac complication [4, 5, 7, 8, 12, 32], with all seven reporting a positive association (Additional file 4: Table S3). The strongest association was shown by Feng et al. who found that a history of MI was associated with a significantly increased odds ratio for cardiac complication following TJA (15.1, 95% Cl 4.1–56.3; *p* < 0.001) [4]. Congestive heart failure and coronary artery disease were found to be predictive risk factors for cardiac complication in two studies [11, 32], with the study by Menendez et al. also identifying an association with peripheral vascular and valvular disease (Additional file 4: Table S3) [11].

### Obesity

Of 15 articles in this series, 8 investigated the effect of obesity on cardiac complication post-TJA (Additional file 4: Table S3) [4, 7, 8, 17, 29, 30, 33, 34]. Feng et al. reported that study participants with BMI ≥ 30 were at greater risk of cardiac complication (OR 2.477; Cl 95% 1.17–5.21; *p* = 0.017) [4]. None of the other seven studies demonstrated a statistically significant relationship between obesity and cardiac complication associated with THA and TKA (*p* > 0.05). Conversely, Anoushiravani et al. and Thornqvist et al. suggested a possible relationship between underweight patients undergoing TJA and cardiac complication; however, a *p* value was not reported (Additional file 4: Table S3) [8, 30].

### Hypertension

The effect of hypertension on risk of cardiac complication was analysed in four studies (Additional file 3: Table S2) [5, 7, 11, 12]. Belmont Jr. et al. reported statistically significant associations for both TKA (OR 4.74; CI 95% 1.04–21.59; *p* = 0.044) and THA (OR 2.59; CI 95% 1.07–6.23; *p* = 0.0341) [5]. Similar findings were demonstrated by Waterman et al. for TKA (OR 2.14; 95% CI 1.30–3.52; *p* = 0.003) and THA (OR 1.82; 95% CI 1.09–3.03; *p* = 0.02) [12]. In contrast, Menendez et al. found that hypertension was not associated with increased risk of cardiac complication (OR 0.9; CI 95% 0.8–1.0; *p* = 0.003) [11], whilst Shah et al. similarly found no effect for this risk factor (Additional file 3: Table S2) [7].

### Diabetes

Four studies examined an association between diabetes and cardiac complication (Additional file 3: Table S2) [5, 7, 8, 11]. Belmont Jr. et al. found that for TKA, diabetes was positively associated with post-operative cardiac complications (OR 2.62; CI 95% 1.41–4.87; *p* = 0.0023); however, the results were not significant for THA [5]. Menendez et al. discussed cardiac complication in relation to complicated and uncomplicated diabetes, with both shown to be associated (OR 1.1; CI 95% 1.1–1.2; *p* < 0.001 and OR 1.2; CI 95% 1.0–1.3; *p* < 0.04, respectively) [11]. No relationship between diabetes and cardiac complication was found by Shah et al. (Additional file 3: Table S2) [7].

### Type of arthroplasty (THA vs. TKA)

Differences between THA and TKA in relation to cardiac complication were examined in four of the studies (Additional file 3: Table S2) [4, 7, 8, 11]. In comparison to TKA, Menendez et al. showed that THA was a better predictor for post-operative cardiac complication (OR 1.3; CI 95% 1.3–1.4; *p* < 0.001) [11]. Shah et al. found increased risk of MI and CA in both THA and TKA populations compared with knee arthroscopy controls (OR 2.61; CI 95% 1.46–4.69; *p* = 0.001 and OR 1.98; CI 95% 1.08–3.62; *p* = 0.03, respectively) (Additional file 3: Table S2) [7].

### Other risk factors

Feng et al. found no difference in cardiac complication risk for TKA and THA, although the authors did identify simultaneous bilateral procedures as a greater predictor of cardiac events than unilateral arthroplasty (OR 1.57; Cl 95% 1.074–2.294; *p* = 0.02) (Additional file 4: Table S3) [4]. Whilst one study reported an odds ratio of 1.1 (CI 95% 1.0–1.2; *p* < 0.006) for the association between renal failure and cardiac complication [11], Shah et al. and Belmont Jr. et al. found no significant association (Additional file 3: Table S2) [5, 7]. Four studies examined the effect of cerebrovascular disease; however, only two studies demonstrated statistically significant results [5, 7, 8, 11]. Menendez et al. and Belmont Jr. et al. concluded that cerebrovascular disease increased the risk of post-TJA cardiac complication (OR 2.3; CI 95%, 2.0–2.6; *p* < 0.001 and OR 2.20; CI 95% 1.02–4.75; *p* = 0.0441 [THA population], respectively) (Additional file 4: Table S3). Four studies also examined chronic pulmonary disease as a risk factor for cardiac complication [5, 7, 8, 11]. Menendez et al. reported an odds ratio of 0.8 (CI 95% 0.7–0.8; *p* < 0.001) for an acute MI, whereas Shah et al. and Belmont et al. did not show an association (Additional file 3: Table S2). Malnutrition, defined as an albumin level < 3.5 g/dL, and adjusted for BMI, was associated with greater risk of cardiac complication in the study by Fu et al. (OR 2.23; CI 95% 1.21–4.12; *p* = 0.010) [33]. Out of three studies examining anaemia and cardiac complication, only Menendez et al. showed statistical significance (OR 1.4; CI 95% 1.3–1.5; *p* < 0.001) [5, 11, 35]. Smoking history was only examined by Shah et al. who found a significant association with cardiac complication in a THA population (OR 2.56; CI 95% 1.34–4.91; *p* = 0.005) [7]. Finally, of the two studies that employed the American Society of Anesthesiologists (ASA) scoring system, only Shah et al. demonstrated an association [5, 7]. In their analysis of the effect of ASA 3 to 4 in both THA and TKA, only the TKA population was significantly predictive of cardiac complication (OR 2.47; CI 95% 1.28–4.74; *p* = 0.007) [7].

## Discussion

The projected rise in hip and knee arthroplasty in the next decade has emphasised the importance in understanding the risks and complications associated with these procedures [4–6]. Myocardial infarction, cardiac arrest, cerebrovascular accidents, deep vein thrombosis, pulmonary embolism, and acute renal failure are major complications known to be associated with THA and TKA [36, 37]. Whilst current literature has extensively evaluated and identified peri- and post-operative complications that can be expected with TJA, there is an overall lack of evidence regarding patient risk factors associated with development of complications, in particular, cardiac complications. A previous systematic review identified risk factors associated with venous thromboembolism in TJA; however, the study by Zhang et al. did not specifically investigate post-operative cardiac complication, therefore necessitating this review [19]. A thorough understanding of these risk factors will allow for better pre-operative care to reduce the incidence of cardiac complication in TJA. The reasons for limiting this systematic review to studies published in 2008 and onwards, as well as those with at least 50% of surgeries occurring since 2008 are two-fold. Firstly, significant improvements in surgical techniques [25, 38, 39], implant technology [39, 40], peri-operative [41] and post-surgical management [40], changes in anaesthetic practices [42–45], and the introduction of ‘fast-track’ hip and knee arthroplasty [42, 44, 46] and same-day bilateral arthroplasty have occurred over the past decade [47]. Secondly, THA and TKA are increasingly performed in older patients as life expectancy increases [14, 39, 41], and those with a greater burden of medical comorbidities including obesity, diabetes, and hypertension [5, 43, 48]. Thus, we focused our investigation to the past decade in order to comprehensively assess current cardiac complications in THA and TKA and relationships to patient risk factors.

In our series, Shah et al. reported the lowest incidence of cardiac complication (0.07%), within a patient group containing no reported risk factors [7]. Conversely, Feng et al. reported the highest complication rate of 3.0%, which is likely attributable to their study population, which contained a greater proportion of coronary artery disease patients [4]. The majority of studies reported incidences of cardiac complication between 0.2 and 0.8%, which is similar to that previously reported in the literature [7]. Of the risk factors analysed, age and history of cardiac disease demonstrated the most consistent relationship with cardiac complication following THA or TKA. The majority of studies examined in the current review reported a positive association with these risk factors and peri- and post-operative cardiac complication (Additional files 3 and 4: Tables S2 and S3) [4, 5, 7, 11, 12, 32]. This highlights the importance of age and history of cardiac disease in the pre-operative screening of THA and TKA patients. Obesity was not identified as a risk factor for cardiac complication post-TJA in this series [7, 17, 29, 33, 34]. These findings are of interest to orthopaedic surgeons who are anticipating a more obese patient demographic for arthroplasty in the future [29, 49]. Interestingly, we report a potential relationship between cardiac complication and underweight patients [8, 30], which warrants further investigation. For additional, lesser-reported risk factors that were analysed in the current review (i.e., bilateral arthroplasty, renal failure, cerebrovascular disease, anaemia, smoking history, and ASA class), the reviewed literature suggests some association. As many of these factors are well-established risk factors for cardiac complication following non-cardiac surgery [50], further research will be necessary to better determine these associations following THA and TKA.

Findings were less clear for other highly reported risk factors such as gender, hypertension, diabetes, and type of arthroplasty, due to greater discrepancy between studies. These inconsistencies were surprising given that gender, hypertension, and diabetes are considered classical cardiovascular risk factors in non-cardiac surgery [13, 50]. For diabetic patients, previous studies have also reported a statistically significant increased risk of MI following arthroplasty; however, these did not meet the inclusion criteria for this review [18, 51]. The inconclusive results in the current review with respect to these particular risk factors may reflect the changes and improvements in arthroplasty previously mentioned, and/or improved management of these risk factors, particularly diabetes and hypertension. However, further investigation is required to better understand the factors contributing to these variances.

Our findings must be considered in the context of the quality of the included studies, together with the wider literature. As evidenced by the results of our appraisal tool (Additional file 2: Table S1), the majority of studies in this series were of moderate quality and multiple limitations were identified that potentially contributed to the discrepancies in results between studies. A commonly encountered limitation in this series was the lack of consistency in study design. For example, although Shah et al. identified significant associations, their study design must be carefully considered for accurate interpretation of data. A knee arthroscopy (KA) population was used as a control cohort with the presumption that KA is a lower risk treatment alternative for osteoarthritis. However, this view is controversial in the current literature [7]. Similarly, in their study of BMI and cardiac complication, Thornqvist et al. included overweight patients (BMI 25 to 30) as a reference for all analysis [8], rather than patients within a normal BMI range as is typically used in the literature. Additionally, although Thornqvist et al. and Anoushiravani et al. report a possible relationship between underweight patients and the risk of cardiac complication in TJA, accurate interpretation is impaired by the absence of a reported *p* value [8, 30]. Furthermore, many of the studies used shared databases, such as the NSQIP and the NIS. Whilst these databases have allowed access to a large volume of data and assisted in developing established pre- and post-operative risk stratifications models, there are limitations when used for analyses. For example, the NSQIP only includes data to 30 days after a surgical procedure and contains limited information regarding several comorbidities [52]. Similarly, the NIS only records inpatient events, does not allow for longitudinal tracking of patients, and does not facilitate distinction between comorbidities from complications [52]. In light of this, not all cardiac events would be identified by studies in this series and some risk factors may be unaccounted for.

To reduce the confounding effect of other disease processes (e.g. rheumatoid arthritis), this review excluded studies that grouped both OA and non-OA patients without adequate sub-analysis. Despite this, all studies included in our series, except four [4, 29, 32, 33], did not specify an indication for TJA and therefore the effect of cofounders cannot be accurately considered. This influences the reliability of the results, which may not adequately reflect the interaction between particular risk factors and cardiac complication. Similarly, many studies did not distinguish between unilateral or bilateral arthroplasty despite different risk profiles of these two procedures [53]. This lack of specificity is a shortcoming of the studies in this series, as well as the current broader literature. Moreover, due to the reliance on national databases for studies in this review, few studies defined their cardiac outcomes (MI, CA, or arrhythmia) to ensure consistency with the current guidelines. Across the various studies, risk factors and outcomes were either poorly defined or defined differently, which contributed to the heterogeneity of the data set and difficulty of conducting a reliable comparative analysis [33].

Additional issues were identified in the broader literature with respect to the effects of many risk factors on peri- and post-operative outcomes. The primary concern encountered with these studies was a lack of further analysis to identify which risk factors were associated with a given complication. This lack of detailed investigation is unfortunate, particularly since in many cases the necessary data was available. Many full-texts that were excluded discussed the relationship between risk factors and increased hospital re-admission, length of stay, major complication or mortality with little or no reference to the individual complications. Although the findings of the current review will help surgeons better understand the risks for patients undergoing TJA, will assist pre-operative counselling of patients, and will allow for better hospital resource allocation, the data does not provide sufficient guide for stratifying patients at greater risk of cardiac complication. It is crucial that future studies provide more robust analysis in order to better determine the association between patient risk factors and post-arthroplasty cardiac complications. Future studies should also investigate various combinations of risk factors, rather than analysing each factor as an independent variable.

One of the major limitations of the current review is the exclusion of potentially relevant studies and risk factors due to the stringent inclusion/exclusion criteria utilised. However, this was necessitated by the lack of specificity in the current literature. Other important risk factors not included in this series include obstructive sleep apnoea and atrial fibrillation. In a study of 530,809 patients, Memtsoudis et al. showed that sleep apnoea was associated with greater odds of cardiac complication (adjusted OR 1.59; CI 95% 1.48–1.71; *p* < 0.001) [54]. With an increasing prevalence of sleep apnoea, this is an important area of future research [54, 55]. Chronic kidney disease has also been shown to be a significant predictor of cardiac complication (OR 5.5; CI 95% 1.68–9.39; *p* = 0.002), as has atrial fibrillation (OR 2.4; CI 95% 2.1–2.8; *p* < 0.0046) [56, 57]. Therefore, it is evident that, whilst this systematic review provides an extensive evaluation on the current literature, there are several other relevant studies, which did not meet our inclusion criteria. The exclusion of foreign language studies constitutes a language bias in this review, whilst there is a potential selection bias for studies utilising the large patient databases. Furthermore, publication bias resulting in only significant results being published and/or being published earlier may result in an over-representation of significant associations between risk factors and cardiac complications.

## Recommendations

With the exception of age and history of cardiac disease, it is apparent that additional risk factors for cardiac complications following TKA and THA require further investigation. Future studies should ensure risk factors, and cardiac complications are defined consistently and measured using validated tools. Currently, the majority of the studies available in the current literature are retrospective in nature. More prospective studies may facilitate better designed cohorts, avoiding the limitations of larger databases and allowing for better conformity among studies. Lastly, for the literature to be applicable to the risk stratification of TKA and THA patients, there is an imperative for more targeted studies that specifically attempt to link risk factors to a cardiac complication (e.g. MI, CA, or arrhythmia). Ultimately, data from such studies can contribute to the development of pre-operative risk stratification models that allow for pre-operative optimisation, and therefore, fewer cardiac complications. Whilst Waterman and colleagues have already attempted this successfully, other risk factors should be further evaluated to make for a more comprehensive predictive tool [12].

## Conclusion

As the rates of THA and TKA continue to rise, a greater understanding about the risk factors for cardiac complication is necessary. From this systematic review, older age, and history of cardiac disease can be considered as the most consistently reported risk factors for cardiac complication in the current literature. Interestingly, this review suggests that obesity may not increase the risk of cardiac complication; however, more robust analysis is required. The current literature is inconclusive for other important risk factors, including diabetes, hypertension, gender, and history of smoking, and these therefore require further investigation. More comprehensive, targeted data can be achieved by using specifically tailored cohorts in which confounders, including arthroplasty indication, unilateral vs. bilateral, and cemented vs. non-cemented prostheses, are adequately controlled for. Ultimately, this data can be used in the development of an extensive pre-operative risk assessment tool that can guide cardiology intervention to minimise cardiac complication post-THA and TKA.

## Additional files

Additional file 1: PRISMA 2009 Checklist. (DOCX 22 kb)

Additional file 2: **Table S1.** Critical Appraisal Questionnaire. (DOCX 45 kb)

Additional file 3: **Table S2.** Summary of results of multivariate analysis of age, gender, type of arthoplasty, diabetes, diabetes, chronic pulmonary disease, renal disease, and hypertension, and relationship to cardiac complication associated with THA and TKA. (DOCX 28 kb)

Additional file 4: **Table S3.** Summary of results of multivariate analysis of obesity, history of cardiac disease, and cerebrovascular disease, and relationship to cardiac complication associated with THA and TKA. (DOCX 36 kb)

## Abbreviations

ACS NSQIP: American College of Surgeons National Surgical Quality Improvement Program
ASA: American College of Anesthesiologists
BMI: Body mass index
CA: Cardiac arrest
CI: Confidence interval
HR: Hazard ratio
KA: Knee arthroscopy
MI: Myocardial infarction
NIS: National Inpatient Sample
OA: Osteoarthritis
OR: Odds ratio
PRISMA: Preferred Reporting Items for Systematic Reviews and Meta-Analysis
RR: Relative risk
THA: Total hip arthroplasty
TJA: Total joint arthroplasty
TKA: Total knee arthroplasty
