# Supplementary material for: Risk factors associated with cardiac complication after total joint arthroplasty of the hip and knee: a systematic review
Source: J Orthop Surg Res. 2019 Jan 11;14:15. doi: 10.1186/s13018-018-1058-9 (PMC6330438; doi:10.1186/s13018-018-1058-9)
Supplement: Supplementary file 2 — Table S1. Critical Appraisal Questionnaire. (DOCX 45 kb) [file 13018_2018_1058_MOESM2_ESM.docx]

Additional file 2: **Table S1** Critical Appraisal Questionnaire.

|  | **Question** | **Waterman ^(12)^** | **Fu ^(33)^** | **Curtis^(32)^** | **Chamieh^(35)^** | **Godoy^(31)^** | **Abdel^(29)^** | **Meller^(17)^** | **Robinson ^(6)^** | **Feng ^(4)^** | **Thornqvist^(8)^** | **Menendez ^(11)^** | **Shah ^(7)^** | **Belmont ^(5)^** | **Anoushira-vani ^(30)^** | | **Meller^(34)^** |
| --- | --- | --- | --- | --- | --- | --- | --- | --- | --- | --- | --- | --- | --- | --- | --- | --- | --- |
|  | **SECTION A: POPULATION** | |  |  |  |  |  |  |  |  |  |  |  |  |  | |  |
| 1 | Is the study population likely to be representative of the whole population? | Y | Y | Y | Y | Y | Y | Y | Y | Y | Y | Y | Y | Y | Y | | Y |
| 2 | Was the non-exposed cohort sourced from the same community or database? | Y | Y | Y | Y | N/A | Y | Y | Y | Y | Y | Y | Y | Y | Y | | Y |
| 3 | Was the exposure ascertained through secure record or structured interview? | Y | Y | Y | Y | Y | Y | Y | Y | Y | Y | Y | Y | Y | Y | | Y |
| 4 | Does the study specify the source of data? | Y | Y | Y | Y | Y | Y | Y | Y | Y | Y | Y | Y | Y | Y | | Y |
| 5 | Was the sample size appropriate or was there a sample size calculation or power analysis performed to determine adequate sample size? | Y | Y | Y | Y | N | Y | Y | Y | N | Y | Y | Y | Y | Y | | Y |
| 6 | Are inclusion and exclusion criteria clearly outlined? | Y | Y | Y | Y | Y | Y | Y | Y | Y | Y | Y | Y | Y | Y | | Y |
| 7 | Is the study population specifically sought for the purpose of the study i.e. no part of a larger shared database? | N | N | N | N | Y | Y | N | N | N | N | N | N | N | N | | N |
|  | **SECTION B: DATA COLLECTION AND METHODOLOGY** | | | | | | | | | | | | | | | | |
| 8 | Is the data range of the data set clearly stated? | Y | Y | Y | Y | Y | Y | Y | Y | Y | Y | Y | Y | Y | Y | | Y |
| 9 | Did the study include a control group? | N/A | Y | Y | Y | N | Y | Y | Y | Y | Y | Y | Y | Y | Y | | Y |
| 10 | Is the research methodology clearly stated? | Y | Y | Y | Y | Y | Y | Y | Y | Y | Y | Y | Y | Y | Y | | Y |
| 11 | Is the data collection methodology clearly stated? | Y | Y | Y | Y | Y | Y | Y | Y | Y | Y | Y | Y | Y | Y | | Y |
| 12 | Is the statistical methodology appropriate? | Y | Y | Y | Y | Y | Y | Y | Y | Y | Y | Y | Y | Y | Y | | Y |
| 13 | Does the study specify that the participants are indicated for TJA for osteoarthritis? | N | Y | Y | N | N | Y | N | N | Y | N | N | N | N | N | | N |
| 14 | Does the study specify between unilateral and bilateral TJA? | Y | N | N | Y | N | Y | N | N | Y | N | Y | N | Y | N | | N |
| 15 | Did the study differentiate between specific cardiovascular complications? | N | N | Y | Y | Y | Y | Y | Y | Y | N | Y | N | Y | N | | Y |
| 16 | Did the study discuss the definitions of myocardial infarction, cardiac arrest and arrhythmia and was this consistent with current guidelines? | N | N | N | Y | Y | Y | N | N | N | N | Y | Y | N | Y | | N |
| 17 | Did the study report a time to follow-up or observational period for which cardiovascular complication can occur? | Y | Y | Y | Y | Y | N | Y | Y | Y | Y | N | Y | Y | N | | Y |
|  | **SECTION C: RESULTS** | | | | | | | | | | | | | | | | |
| 18 | Are the outcomes clearly stated and discussed in relation to the data collection? | Y | Y | Y | Y | Y | Y | Y | Y | Y | Y | Y | Y | Y | Y | | Y |
| 19 | Does the study report findings in relation to original aims? | Y | Y | Y | Y | Y | Y | Y | Y | Y | Y | Y | Y | Y | Y | | Y |
| 20 | Are the participants baseline demographics detailed for all groups? | Y | Y | Y | Y | N | Y | Y | Y | Y | Y | Y | Y | Y | Y | | Y |
| 21 | If containing THA and TKA patients, does the study differentiate between these two populations i.e. not made into a single cohort. | Y | N/A | N/A | N/A | N/A | N/A | N/A | Y | N | N | Y | Y | Y | Y | | N/A |
| 22 | If comparisons were made between THA and TKA were the two populations similar? | Y | N/A | N/A | N/A | N/A | N/A | N/A | Y | N/A | N/A | Y | Y | Y | Y | | N/A |
| 23 | Was the outcome assessed by reliable means i.e. record linkage? | Y | Y | Y | Y | Y | Y | Y | Y | Y | Y | Y | Y | Y | Y | | Y |
| 24 | Were confidence intervals provided with odds/risk/hazard ratios? | Y | Y | Y | Y | N/A | Y | Y | Y | Y | Y | Y | Y | Y | Y | | Y |
| 25 | Was there complete follow-up or a percentage greater than 95% follow-up? | Y | Y | Y | Y | Y | Y | Y | Y | Y | Y | Y | Y | Y | Y | | Y |
|  | **SCORE** | **20/24** | **19/23** | **20/23** | **21/23** | **16/21** | **22/23** | **19/23** | **21/25** | **20/24** | **18/24** | **22/25** | **21/25** | **22/25** | | **20/25** | **19/23** |

**Table S1** Quality assessment of studies included in the systematic review. Scores calculated from the number of Y answers divided by the total number of questions excluding all that are not applicable. Y: Yes, N: No, N/A: not applicable. The scores presented in the tables represent the average between two of the authors (YE and IJ) who each performed the critical appraisal independently.
