# Supplementary material for: Risk factors associated with cardiac complication after total joint arthroplasty of the hip and knee: a systematic review
Source: J Orthop Surg Res. 2019 Jan 11;14:15. doi: 10.1186/s13018-018-1058-9 (PMC6330438; doi:10.1186/s13018-018-1058-9)
Supplement: Supplementary file 3 — Table S2. Summary of results of multivariate analysis of age, gender, type of arthoplasty, diabetes, diabetes, chronic pulmonary disease, renal disease, and hypertension, and relationship to cardiac complication associated with THA and TKA. (DOCX 28 kb) [file 13018_2018_1058_MOESM3_ESM.docx]

Additional file 3: **Table S2** Summary of results of multivariate analysis of risk factor and relationship to cardiac complication associated with THA and TKA.

| **Author** | **Odds Ratio** | **Age** | **Gender** | **THA vs TKA** | **Diabetes** | **Chronic pulmonary disease** | **Renal Failure** | **Hypertension** |
| --- | --- | --- | --- | --- | --- | --- | --- | --- |
| **Belmont Jr (5)** | Development of adverse cardiac events post-operatively | ≥ 80: TKA: OR, 27.95, CI 95% 2.01-388.93; p=0.0016. THA: OR, 3.72, CI 95% 1.53-9.06; p=0.0001. | N/S* | N/R | TKA: OR, 2.62, CI 95% 1.41-4.87; p=0.0023 | N/S* | N/S* | TKA: OR, 4.74, CI 95% 1.04-21.59; p=0.0440 THA: OR, 2.59, CI 95% 1.07-6.23; p=0.0341 |
| **Shah (7)** | Compared to knee arthroscopy control: MI/CA | Mean Age:  THA: OR, 1.06, CI 95% 1.03-1.09; p<0.001 TKA: OR, 1.03, CI 95% 1.00-1.06; p=0.04 | N/S* | THA: OR, 2.61, CI 95% 1.46-4.69; p=0.001 TKA: OR, 1.98, CI 95% 1.08-3.62; p=0.03 | N/S* | N/S* | N/S | N/S* |
| **Menendez (11)** | Rate of Acute MI | Reference: <45 years 45-64: OR, 4.4, CI 95%: 2.6-7.4; p<0.001. 65-84: OR, 6.5, CI 95%: 3.8-11.0; p<0.001. >85: OR, 9.4, CI 95%: 5.5-16.1; p<0.001 | Reference: Female Male: OR, 1.4, CI 95%: 1.4-1.5; p<0.001 | THA (reference: TKA):  OR, 1.3, CI 95%: 1.3-1.4; p<0.001 | Complicated Diabetes:  OR, 1.1, CI 95%: 1.1-1.2; p<0.001  Uncomplicated Diabetes:  OR, 1.2, CI 95%: 1.0-1.3; p<0.04 | OR, 0.8, CI 95%: 0.7 - 0.8; p<0.001 | OR, 1.1, CI 95%: 1.0-1.2; p<0.006 | OR, 0.9, CI 95%: 0.8-1.0; p=0.003 |
| **Feng (4)** | Cardiac complication | N/S | N/S | N/S | N/R | N/R | N/R | N/R |
| **Waterman (12)** | Postoperative cardiac event | **≥80:**  TKA: OR, 1.85, 95% CI, 1.23-2.79; p=0.003).  THA: OR, 4.39, 95% CI, 2.29-6.61; p<0.001. | N/R | N/R | N/R | N/R | N/R | TKA: OR, 2.14, 95% CI, 1.30-3.52; p=0.003)  THA: OR, 1.82, 95% CI, 1.09-3.03; p=0.02 |
| **Robinson (6)** | Cardiac complication | N/R | THA:  Female: OR, 0.54, 95% CI, 0.40-0.75; p<0.001  **MI:** Male: OR, 1.84, 95% Cl, 1.34-2.52; p<0.001  TKA:  Female: OR, 0.59, 95% CI, 0.45-0.76; p <0.001 **MI:** Male: OR, 1.71, 95% Cl, 1.31-2.22; p<0.001 | N/R | N/R | N/R | N/R | N/R |

**Table S2** Associations between age, gender, diabetes, chronic pulmonary disease, renal failure, hypertension and type of arthroplasty (THA vs. TKA) and a cardiac complication. Only significant values are included (p < 0.05). Hazard ratios and relative risks that were performed by some studies were not included in this table due to lack of significance. N/R = Not reported; MI: myocardial infarction; CA: cardiac arrest; N/S: not statistically significant result. * Studies did have significant bivariate analysis, but multivariate analysis was not found to be significant.
