# Supplementary material for: Risk factors associated with cardiac complication after total joint arthroplasty of the hip and knee: a systematic review
Source: J Orthop Surg Res. 2019 Jan 11;14:15. doi: 10.1186/s13018-018-1058-9 (PMC6330438; doi:10.1186/s13018-018-1058-9)
Supplement: Supplementary file 4 — Table S3. Summary of results of multivariate analysis of obesity, history of cardiac disease, and cerebrovascular disease, and relationship to cardiac complication associated with THA and TKA. (DOCX 36 kb) [file 13018_2018_1058_MOESM4_ESM.docx]

Additional file 4: **Table S3** Summary of results of multivariate analysis of risk factor and relationship to cardiac complication associated with THA and TKA.

| **Author** | **Odds Ratio** | **Obesity** | **History of cardiac disease** | **Cerebrovascular disease** |
| --- | --- | --- | --- | --- |
| **Shah(7)** | Compared to knee arthroscopy control: MI/CA | N/S | THA: OR, 2.88, CI 95% 1.31-6.33; p=0.01 TKA: OR, 1.67, CI 95% 0.66-4.23; p=0.28 | N/S |
| **Feng(4)** | Cardiac complication | BMI ≥ 30 OR, 2.477, Cl 95% 1.17-5.21; p=0.017 | History of MI: OR, 15.1, 95% Cl 4.1-56.3, p>0.001  CAD:  CAD with REVASC: OR, 11.3, 95% Cl 5.7-23.1, p<0.001  CAD without REVASC: OR, 11.0, 95% Cl 7.7-18.9, p<0.001 | N/R |
| **Meller^ϒ^(34)** | Acute MI | N/S | N/R | N/R |
| **Meller^ϒΦ^(17)** | Acute MI | N/S | N/R | N/R |
| **Anoushiravani(30)** | Cardiac complication | N/S* | N/R | N/R |
| **Fu(33)** | Postoperative cardiac event. | N/S | N/R | N/R |
| **Abdel(29)** | N/R | N/S | N/R | N/R |
| **Curtis(32)** | N/R | N/R | **CHF:** (OR 4.81, 95% CI 1.90-12.16, p = 0.001) | N/R |
| **Waterman (12)** | Postoperative cardiac event. | N/R | TKA: OR, 4.03, 95% CI, 2.08-7.80; p<0.001  THA: OR, 3.74, 95% CI, 1.77-7.88; p<0.001 | N/R |
| **Robinson(6)** | Cardiac complication | N/R | N/R | N/R |
| **Menendez (11)** | Acute MI | N/R | CHF: OR: 2.6, CI 95%: 2.4-2.8; p<0.001  Valvular disease: OR: 1.2, CI 95% 1.1-1.3; p<0.001  PVD: OR: 1.5, CI 95%: 1.4-1.7; p<0.001  CAD: OR: 4.9, CI 95%: 4.6-5.2; p<0.001 | OR, 2.3, CI 95%: 2.0-2.6; p<0.001 |
| **Belmont Jr(5)** | Development of adverse cardiac events postoperatively | N/R | TKA: OR, 4.46, CI 95% 2.29-8.67; p<0.0001 THA: OR, 2.80, CI 95% 1.51-5.20; p=0.0011  CHF: N/S *  PVD: N/S * | THA: OR, 2.20, CI 95% 1.02-4.75; p=0.0441 |

**Table S3** Associations between obesity, history of cardiac disease and cerebrovascular disease and cardiac complication. Only significant values are included (p < 0.05). Hazard ratios and relative risks that were performed by some studies were not included in this table due to lack of significance. N/R = Not reported; MI: myocardial infarction; CA: cardiac arrest; CAD: coronary artery disease; REVASC: revascularisation; CHF: congestive heart failure; PVD: peripheral vascular disease; N/S: not statistically significant result. * Studies did have significant bivariate analysis, but multivariate analysis was not found to be significant. ϒStudies used hazards ratios rather than odds ratios for their multivariate analysis. **^Φ^** In this study, total joint arthroplasty is THA.
